# Vagal Flexibility Mediates the Association Between Resting Vagal Activity and Cognitive Performance Stability Across Varying Socioemotional Demands

**DOI:** 10.3389/fpsyg.2020.02093

**Published:** 2020-09-09

**Authors:** Derek P. Spangler, Jared J. McGinley

**Affiliations:** ^1^Human Research and Engineering Directorate, U.S. Army Research Laboratory, Aberdeen, MD, United States; ^2^Department of Psychology, Towson University, Towson, MD, United States

**Keywords:** heart rate variability, vagal activity, cognition and emotion, stress, flexibility

## Abstract

Vagal flexibility describes the ability to modulate cardiac vagal responses to fit a dynamic range of challenges. Extant theory on vagal function implies that vagal flexibility is a mediating mechanism through which resting vagal activity, a putative individual difference related to self-regulation, affects adaptive behavior and cognition. Nevertheless, little research has directly tested this hypothesis, thereby leaving fundamental mechanisms of vagal function and adaptability unclear. To this end, 47 healthy subjects completed a 5 min baseline followed by Stroop tasks combined with concurrent auditory distractors. There were four different Stroop task conditions that varied the social and emotional content of the auditory distractors. Electrocardiogram was continuously recorded to assess vagal responses to each condition as heart rate variability [root mean square of successive differences (RMSSDs)] reactivity. Vagal flexibility significantly mediated the association between resting vagal activity and stability of inhibition performance (Stroop interference) scores. In particular, higher resting RMSSD was related to higher standard deviation of RMSSD reactivity scores, reflecting greater differences in RMSSD reactivity between distractor conditions (i.e., greater vagal flexibility). Greater vagal flexibility was in turn related to more stability in Stroop interference across the same conditions. The mean of RMSSD reactivity scores across conditions was not significantly related to resting RMSSD or stability in Stroop performance, and mean RMSSD reactivity did not mediate relations between resting RMSSD and stability in Stroop performance. Overall, findings suggest that vagal flexibility may promote the effects of resting vagal activity on stabilizing cognitive inhibition in the face of environmental perturbations.

## Introduction

Self-regulation describes the regulation of cognition, behavior, and physiology to fit changing situational demands and inherently involves change (Cole et al., 2019). At the physiological level, self-regulation is believed to be facilitated by the vagus nerve (Porges, 2007; Thayer and Lane, 2009). Much research on this topic has focused on tonic cardiac vagal activity, which is often measured with resting vagally mediated heart rate variability (HRV^1^). Resting HRV is believed to be a trait indicator of physiological self-regulation capacity that supports effective behavioral and cognitive regulation as well as superior health and well-being (Thayer et al., 2009; Kemp and Quintana, 2013).

A longstanding question is how a static snapshot of vagal activity at rest contributes to the adaptive regulation of cognition and behavior across varying challenges. According to the polyvagal theory and neurovisceral integration model, the link between resting vagal activity and state behavior is mediated by *vagal flexibility*^2^ *–* the dynamic regulation of vagal responses to fit different contexts (Porges et al., 1996; Thayer and Lane, 2000). Specifically, tonic vagal activity is theorized to reflect the capacity for modulating vagal activity to meet varying environmental demands (Thayer and Lane, 2009). Such modulation (i.e., vagal flexibility) is thought to more directly represent the state dynamics of self-regulation, namely, shifts in cardiac output and prefrontal cortex (PFC) activation across challenges (Porges, 1995a, b; Thayer et al., 2012). These shifts tune metabolic and attentional responses to fit the task at hand, thus supporting adaptive cognitive performance (Hockey, 1997; Thayer and Lane, 2000). In other terms, vagal flexibility reflects adaptability of the vagal “brake.” Vagal flexibility may serve as the state-based mechanism through which individuals with high resting vagal activity promote adaptive cognition/behavior to meet changing situational demands.

Although some tenets of the polyvagal theory (e.g., Monteiro et al., 2018), and to a lesser extent neurovisceral integration (e.g., Jennings et al., 2015), have been challenged, the theories’ claims about the role of vagal flexibility in cognition and emotion regulation are not falsified and remain influential. There is much evidence for a relation between vagal activity and inhibition of inappropriate responses (Jennings, 1992; Thayer and Lane, 2000). Of note, higher resting HRV and greater phasic HRV suppression have been associated with superior cognitive inhibition, measured as weaker interference on Stroop and Flanker tasks (Johnsen et al., 2003; Mathewson et al., 2010; Spangler and Friedman, 2017).

The notion that vagal flexibility mediates the link between resting HRV and adaptive cognition has not been robustly tested. One reason for this gap is that past studies examining HRV and performance have not directly tested the mediational effect (e.g., Duschek et al., 2009; Park et al., 2014). Another reason for this gap is that prior research has relied on static (e.g., mean-based) rather than dynamic measures that reflect variability in phasic vagal activity and cognition across multiple contexts (Nesselroade and Ram, 2004). Static metrics, which reflect mean activity or activity at a single time point, are too simple to capture the situationally appropriate regulation of vagal function and cognition that has been emphasized in extant vagal theory. Of note, many studies have purported to measure vagal flexibility (i.e., adaptability of the vagal “brake”) with reactivity difference scores (task HRV-baseline HRV), thus only examining vagal responses to a single challenge (e.g., Butler et al., 2006; Duschek et al., 2009) or as mean vagal reactivity across a few challenges (Muhtadie et al., 2015; Human and Mendes, 2018). These studies fail to capture how vagal responses are flexibly varied (or adjusted) to fit different contexts.

Because adaptability putatively relies on modulating vagal responses to fit a dynamic range of contexts (Porges, 2007; Thayer and Lane, 2000), studies should operationalize vagal flexibility as intraindividual variability (IIV) in vagal reactivity across different tasks. Broadly, IIV is an influential construct describing the magnitude of dynamic, within-person change in psychophysiological activity such as vagal reactivity or performance (Nesselroade and Ram, 2004; MacDonald et al., 2009). Relative to mean metrics, the degree of IIV is thought to better indicate regulatory processes that maintain homeostasis or stability at one end of the continuum (corresponding to low IIV scores) and lability or instability at the other end (corresponding to high IIV scores) (Ram and Gerstorf, 2009). In accord with this perspective, IIV in vagal reactivity may effectively reflect the dynamic regulatory processes that maintain stability in cognition despite environmental perturbations.

Similarly, prior research on vagal–cognition relations is also limited because it has focused on mean performance as opposed to IIV in performance – the degree to which cognition is more (or less stable) in the same individual over time. Relative to mean performance, IIV in performance is thought to better reflect the dynamic frontal lobe and inhibitory/attentional control functions theoretically linked to adaptive behavior and vagal activity (Stuss et al., 2003; Weissman et al., 2006; MacDonald et al., 2009). High resting HRV has been related to higher levels of performance consistency, a specific IIV metric reflecting the degree to which performance does or does not vary trial-to-trial during a single task. Consistency is often measured as the standard deviation (SD) of response times (RT), and lower consistency (i.e., higher SD) is thought to reflect interruptions to cognition caused by endogenous factors (e.g., mind-wandering; Hultsch and MacDonald, 2004; Unsworth, 2015; Williams et al., 2016; Spangler et al., 2018b).

Vagal theories emphasize adaptability in the face of environmental challenges as opposed to endogenous factors like mind-wandering. Thus, studies of HRV should examine a different IIV performance metric that reflects whether performance is stable across different exogenous task demands (Hultsch and MacDonald, 2004). This metric, which we term performance stability^3^, can be measured with the SD of accuracy or RT scores across multiple tasks (Hines et al., 2016; Jones et al., 2018). Lower SD of performance scores across tasks index greater performance stability. Lower levels of performance stability can be adaptive, such as when response inhibition is rapidly decreased from neutral to physically threatening contexts, in order to promote survival-enhancing behavior (Nesse, 2005). In most daily settings, motivational (social, emotional) stimuli do not pose physical threat but instead operate as salient distractors (Dolcos and McCarthy, 2006). It is adaptive in these situations to exhibit more stability in performance despite the flux of motivational stimuli (Wacker, 2018). High-performance stability relies on dynamic top-down control functions that inhibit the effects of environmental perturbations on cognition (Stuss et al., 2003; Armbruster et al., 2012; Iordan et al., 2013). As such, performance stability may represent a capacity for robust cognitive processes that is not reflected by mean levels of performance (MacDonald et al., 2009; Morgan et al., 2011). In line with the neurovisceral integration model, the dynamic regulation giving rise to performance stability may be proxied by the degree of task-related shifts in vagal responses (i.e., vagal flexibility; Thayer et al., 2012).

Only one study has investigated a dynamic individual difference metric of vagal flexibility alongside performance stability across changing tasks (Spangler et al., 2018a). Here, individuals with higher vagal flexibility across multiple conditions (rest, recovery, shooting conditions) exhibited: (i) higher resting HRV and (ii) less stability in response inhibition performance – stronger increases in friendly fire from low to high threat (vibration to shock) in a shoot/no-shoot task. Vagal flexibility was inferred to support the effect of high resting vagal activity on the adaptive lowering of inhibitory control during physical danger (painful shock), in turn promoting the expression of survival-enhancing defensive responses (Nesse, 2005).

## Current Study

In order to more fully test the role of vagal flexibility in adaptive cognition, the current study examined vagal flexibility as a mediating mechanism linking tonic resting vagal activity and performance stability. Importantly, the present study operationalized vagal flexibility and performance stability with IIV in HRV and performance across different contexts, thereby capturing theoretically emphasized dynamics in vagal activity and cognition that most studies have ignored. The present study expanded on our prior study (Spangler et al., 2018a) in three ways. First, rather than examine adaptive instability in performance due to painful stimuli, we examined adaptive stability in performance amid innocuous motivational distractors. Second, distractors were varied on emotional and social dimensions during the cognitive task, allowing us to link responses to task demands that are central to vagal theories (Thayer and Lane, 2000; Porges, 2007). Third, unlike prior studies, vagal flexibility was tested as a mediator of the correlations of resting vagal activity to both mean performance and performance stability. In line with strong links between vagal activity and inhibitory control, we focused on stability in cognitive inhibition as our metric of performance stability, which was measured as the degree of IIV in Stroop RT interference across different task conditions (MacLeod, 1991; Thayer and Lane, 2000). Lower levels of IIV indicated greater stability in inhibition performance. Mean inhibition performance was also examined, as mean and IIV metrics convey unique information about neurocognitive function (Spangler et al., 2018b).

The present study utilized a novel experimental paradigm in which auditory distractors were integrated into a visual Stroop task. Motivational demands were manipulated across four conditions, which varied in the degree to which auditory stimuli were emotional and social in nature. We investigated mean performance as the mean of interference scores (Interference_MEAN_) and performance stability as the SD of interference scores (Interference_SD_) across the socioemotional conditions. We adjusted each condition-level HRV score by a preceding baseline and then computed the SD of the reactivity scores (Reactivity_SD_) to index vagal flexibility. To clarify this novel metric relative to a more traditional flexibility measure, we also examined the mean of the reactivity scores (Reactivity_MEAN_; Muhtadie et al., 2015).

In line with theory, higher resting vagal activity should support the adaptive regulation of psychophysiological process to fit different distractor task demands (indexed by greater vagal flexibility), in turn supporting greater performance stability as well as better mean performance across environmental perturbations (Porges, 2007; Ram and Gerstorf, 2009). It was thus hypothesized that greater vagal flexibility (higher Reactivity_SD_) would mediate the associations between higher resting vagal activity (higher resting HRV) and greater stability in inhibition scores (lower Interference_SD_). It was also predicted that greater vagal flexibility (higher Reactivity_SD_) would mediate the relation between higher resting vagal activity (higher resting HRV) and higher mean inhibition performance (lower Interference_MEAN_). Relative to traditional metrics reflecting the degree of vagal reactivity and mean performance, vagal flexibility and performance stability likely better index the dynamic neural regulation underlying goal-directed cognition. We thus predicted that the mediational effect of vagal flexibility would be stronger than that of mean reactivity. Similarly, we also hypothesized that vagal flexibility’s mediational effect would be stronger when estimating performance stability relative to mean performance.

## Materials and Methods

### Subjects

Subjects were 49 undergraduate students from a Mid-Atlantic university (mean age = 19.47, *SD* = 1.44; 65.3% female). All subjects were volunteers who received course credit for participation. All study procedures were approved by the university’s institutional review board. As described below, two outliers were removed, which left 47 subjects (mean age = 19.51, *SD* = 1.46; 66% female) in our analysis. Eligibility requirements for the study were right-handedness, nonsmoking, and no history of cardiovascular or neurological disease or cardiac arrhythmias. Subjects were also asked to abstain from alcohol use for 24 h, caffeine consumption for 6 h, eating for 2 h, and vigorous exercise for 2 h prior to the study session. Sample size was determined with a power analysis for the mediational indirect effect (i.e., the focal hypothesis of the article) using the MedPower application (Kenny, 2017). Based on (1) power of 0.8, (2) α of 0.05, and (3) path a and b correlations of 0.4 (effect sizes consistent with prior studies; e.g., Thayer et al., 2009), a necessary sample size of *N* = 59 was estimated. Because the present analyses included 47 subjects, these analyses were slightly underpowered.

### Materials

#### Socioemotional Distractor Stimuli

The International Affective Digitized Sounds (IADS; Bradley and Lang, 2007) were used as distractors during the Stroop tasks. All IADS sounds were 6 s in duration. The IADS sets are scored on a 1–9-point Likert scale for arousal and valence dimensions. Five members of the experimental team rated each IADS item on preselected criteria. Specifically, the raters were provided with the following instructions: “Please categorize these files into 4 different categories. These categories will be (1) high social–high emotion (e.g., a person yelling while being murdered), (2) high social–low emotion (e.g., the sound of a crowd murmuring in the background, (3) high emotion–low social (e.g., the sound of a gunshot), and (4) low social, low emotion (e.g., the sound of a gentle breeze). I want you to listen to sounds and decide if they fall into these categories, although many will not so don’t put them into a category if they don’t fulfill the criteria. Basically, you want to decide if there is a social element (i.e., human voices); this could be a scream, talking, sounds of sex, etc. You next want to describe if there is an emotional component; this will be decided on whether or not the sounds elicit an emotion for you.”

The audio clips were then cross-referenced and were considered acceptable for use in the study if four of the five raters selected a clip for a given category. Using this approach, 32 distractor sounds were selected for the study (eight for each category). Four socioemotional conditions were created based on a combination of these criteria and rankings: low emotion-low social (LELS), high emotion-low social (HELS), low emotion-high social (LEHS), high emotion-high social (HEHS). For more information on the selected sounds (see Supplementary Appendix A).

#### Stroop + Socioemotional Distractors

During each of the socioemotional conditions, the aforementioned distractor sounds were played during a computerized version of Stroop task, which is commonly used to measure cognitive inhibition (Stroop, 1935). For the Stroop tasks, subjects were instructed to indicate the color of printed “color” words presented on the computer monitor. Word color responses were made with a keyboard press on one of the following numerical keys (2 = “blue,” 4 = “red,” 6 = “green,” 8 = “yellow”). In congruent trials, the colors were consistent with the word (e.g., the word “blue,” presented in blue coloring), and incongruent included color words presented in a different color ink (e.g., the word “blue” presented in yellow coloring). There were 64 trials in each of the four conditions (32 incongruent, 32 congruent). Unlike typical versions of the Stroop, each Stroop word (each trial) was presented simultaneously with one of the aforementioned distractor sounds from the IADS (Bradley and Lang, 2007). Specifically, each sound started 1,000 ms before the presentation of the Stroop word. Both the sound and Stroop word disappeared simultaneously and moved to the next trial once a subject made a response to the word. If a response was not made within 5 s of Stroop word onset, the next trial was presented. The intertrial interval was 1,000 ms. Distractor sounds that were paired with Stroop words were randomly selected from a pool of eight sounds, with each sound being presented eight times during the condition (Supplementary Appendix A). As such, each condition (LELS, LEHS, HELS, HEHS) played sounds from a single socioemotional category, such that conditions differed only based on the category of sounds.

#### Physiological Recording Equipment

A modified lead-II electrocardiogram (ECG) was used to measure cardiac activity. Two disposable, pre-gelled, stress-testing electrodes were attached to the thorax. The ECG signal was transmitted to an ECG100C amplifier (BIOPAC Systems, Inc., Goleta, CA, United States), and then interfaced through an MP150 acquisition system and transmitted to a Dell personal computer in an adjacent room. Data were continuously recorded (sampling rate = 2,000 Hz), and R-spikes were detected with the AcqKnowledge 4.4.2 software (BIOPAC Systems, Inc.). In the process of identifying artifacts in the ECG data, two levels of artifact correction were employed. At the first level, researchers manually corrected mislabeled R-spikes in AcqKnowledge. However, we also expected that there would still be some user error (i.e., misidentified peaks). Therefore, as needed (<1% data), the RR series was additionally corrected for artifacts with Kubios HRV Analysis Software v3.0 (Tarvainen et al., 2014).

### Procedure

**FIGURE 1:**
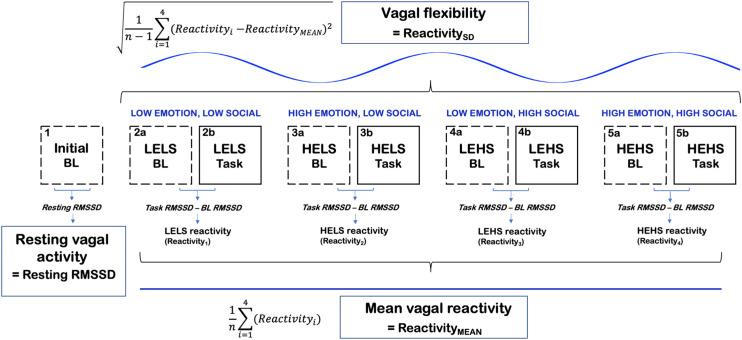
A conceptual figure of the study’s procedure and calculation of vagal [RMSSD] metrics. LELS, low emotion, low social; HELS, high emotion, low social; LEHS, low emotion, high social; HEHS, high emotion, high social.

Each experimental session took place in a sound-attenuated, temperature-controlled, research room; the participants were also provided noise-canceling headphones for the duration of the study. After being connected to physiological equipment and completing health history information, the participants completed a 5 min resting baseline period in which they were instructed to sit quietly and view a series of neutral pictures from the International Affective Picture System (Lang et al., 1997a). Sixty pictures ranked medium on valence (between 4 and 5) and low on arousal (<4) were selected for the baseline images. The baseline was then followed by a practice Stroop period (16 trials). Next, subjects completed the dual task composed of the Stroop and auditory distractors. Conditions were quasi-counterbalanced so that a 2 min baseline always preceded the four different task conditions of the Stroop + auditory distractor task (Figure 1). This method ensured that a high-emotion condition would not directly follow the other high-emotion condition and that a high-social condition would not follow the other high-social condition.

### Data Reduction

Cognitive inhibition was measured with Stroop interference, operationalized as the mean RT of incongruent trials minus the mean RT of congruent trials (incongruent RT-congruent RT) (e.g., Richeson et al., 2003; Larson et al., 2009). Separate interference scores were computed for each of the four socioemotional conditions (LELS, HELS, LEHS, HEHS). Serving as our metric of inhibition performance stability, Interference_SD_ was computed as the SD of condition-level interference scores. Relatively lower levels of Interference_SD_ indexed greater performance stability. Interference_MEAN_ (mean inhibition performance) was calculated as the average of the four condition-level Stroop interference scores. Prior studies have shown that Stroop interference is robustly evidenced by RT differences between congruent and incongruent trials (our approach described above), whereas analogous differences in accuracy data are often weaker (MacLeod, 1991, 1998; MacKay et al., 2004; Cothran and Larsen, 2008). In line with these studies and with the convention of using Stroop RT interference for indexing inhibition (e.g., Miyake et al., 2000), we focus on RT-based as opposed to accuracy-based interference metrics. For completeness, we also present findings using Stroop interference metrics based on accuracy scores (proportion of correct responses for incongruent trials - proportion of correct responses for congruent trials) in Supplementary Materials. None of the accuracy interference effects were significant (*p* > 0.05).

Interbeat intervals from sequential R-spikes were extracted from the ECG signal. Cardiac vagal activity was estimated using well-established HRV metrics, which were quantified from each IBI time series with Kubios HRV Analysis Software v3.0 (Tarvainen et al., 2014). Our primary metric of cardiac vagal activity was the root mean square of successive differences (RMSSDs; ms) in interbeat intervals (Kleiger et al., 1992). Vagal activity was also computed as high-frequency HRV (HF-HRV; power [ms^2^] in 0.15–0.4 Hz) with autoregressive spectral analysis (Montano et al., 1994). We focus on results for RMSSD because, relative to spectral estimates like HF-HRV, RMSSD has been reported to be more statistically reliable and less influenced respiratory confounds (Penttilä et al., 2001; Kuss et al., 2008). Furthermore, RMSSD as a “model-free” metric is less reliant on assumptions of stationarity, which are rarely met in most psychophysiological studies (Kalinichenko et al., 2008). From the spectral analyses, the peak frequency of the HF band (HF-peak) was also extracted as a proxy for respiration rate (Thayer et al., 2002).

The computation of primary vagal metrics is summarized in Figure 1. Tonic vagal activity was measured as RMSSD during the initial 5 min resting baseline that occurred prior to all of the Stroop tasks and their corresponding baselines. We chose the initial baseline to index resting vagal activity because this period was less likely to be confounded by stress compared to the task baselines that occurred later in the procedure. Vagal reactivity scores were computed as RMSSD reactivity where we subtracted each preceding baseline RMSSD score from the corresponding task RMSSD score (task–baseline). Larger negative values on RMSSD reactivity scores conveyed greater decreases in vagal activity in response to the task condition, whereas larger positive values indicated greater increases in vagal activity. The degree of vagal flexibility was computed as the SD of the RMSSD reactivity scores (denoted as Reactivity_SD_). Higher Reactivity_SD_ scores indexed relatively greater differences in vagal responses between the four socioemotional conditions (i.e., greater vagal flexibility). Reactivity_MEAN_ (mean vagal reactivity) was computed as the average of the four condition-level RMSSD reactivity scores (Muhtadie et al., 2015).

### Statistical Analysis

Prior to analyses, assumptions of normality for all variables were tested with Shapiro–Wilk tests (Shapiro and Wilk, 1965). As needed, metrics were natural logarithm (ln) transformed to correct for positive skew. Specifically, we ln transformed (1) resting RMSSD (denoted as resting lnRMSSD) and (2) Reactivity_SD_ (denoted as lnReactivity_SD_). Two outlier cases (>3 SD) were excluded, leaving 47 subjects in the statistical analyses. Specifically, one outlier in Interference_SD_ and another outlier in lnReactivity_SD_ were removed. For the analyses of variance (ANOVAs) only, vagal flexibility was calculated by binning participants into one of three groups after a tertile split of Reactivity_SD_ (low, medium, high). For all other correlational and mediational analyses, vagal flexibility was treated as a continuous variable.

In order to test effects of socioemotional conditions on Stroop interference, we conducted a 2 (congruency: congruent, incongruent) × 2 (emotion: low, high) × 2 (social: low, high) × 3 (vagal flexibility: tertiles 1–3) mixed ANOVA on Stroop RT. Similarly, socioemotional effects on RMSSD reactivity were tested with a 2 (period: baseline, task) × 2 (emotion: low, high) × 2 (social: low, high) × 3 (vagal flexibility: tertiles 1–3) mixed ANOVA on lnRMSSD scores (natural logarithm transformed to reduce its positive skew). We tested interactions between vagal flexibility and the task conditions in order to elucidate how patterns of intercondition change in performance/vagal reactivity varied as a function of vagal flexibility. This ANOVA approach clarified the directional changes in performance/vagal reactivity that might drive the broader association between vagal flexibility and performance stability.

Pearson correlation coefficients tested associations among RMSSD metrics (resting lnRMSSD, Reactivity_MEAN_, lnReactivity_SD_), as well as the relations between these RMSSD metrics and interference measures (Interference_MEAN_, Interference_SD_; Spangler et al., 2018a). We re-ran the RMSSD–interference correlations as partial Pearson correlations coefficients (PCCs) that adjusted for sex, age, body mass index (BMI), and respiration rate (as proxied by HF-peak) – variables that potentially confound relations involving HRV (Grossman et al., 1991; Reardon and Malik, 1996; Molfino et al., 2009; Koenig and Thayer, 2016). For each PCC, an HF-peak measure that was analogous to the HRV metric was entered as a covariate, in order to account for respiration as a confound for that given correlation. For example, PCCs including lnReactivity_SD_ (vagal flexibility) corrected for the SD of HF-peak estimates across conditions (HF-peak_SD_).

In order to test whether vagal flexibility mediated the relation between resting vagal activity and performance stability, we utilized the product of the coefficients and Monte Carlo methods for estimating and testing the indirect effect (MacKinnon et al., 2004). This approach involved conducting three separate regression models. In Model 1, we estimated the total effect of resting HRV on Interference_SD_ before accounting for vagal flexibility (path c). Here, we adjusted for the same covariates as when testing correlations between resting HRV and performance (age, BMI, sex, resting HF-peak). In Model 2, we estimated vagal flexibility with resting HRV adjusting for the same covariates (path a). Model 3 had the same form as Model 1, except that vagal flexibility (mediator) was added as a term to Model 1 (model testing path c) This model yielded the direct effect of resting HRV on Interference_SD_ accounting for vagal flexibility (path c’), as well as the unique effect of vagal flexibility on performance (path b). The mediational effect of vagal flexibility (i.e., indirect effect of resting HRV) was estimated with the product of path a and path b coefficients (a ^∗^ b). The indirect mediational effect was statistically tested against zero using an approximate Bayesian Monte Carlo method (10,000 simulations) (Imai et al., 2010). In order to test whether vagal flexibility fully (or partially) mediated the relation between resting HRV and performance, the statistical significance of path c and that of c’ were compared. For example, if the resting HRV–Interference_SD_ association was significant but became nonsignificant when controlling for Reactivity_SD_, then full mediation would be suggested.

Separate models of the same form were used to examine (i) whether vagal flexibility mediated the relations between resting HRV and Interference_MEAN_ and (ii) whether mean vagal reactivity mediated the relations of resting HRV to performance stability and mean performance.

## Results

### Task Effects on Performance and HRV

**FIGURE 2:**
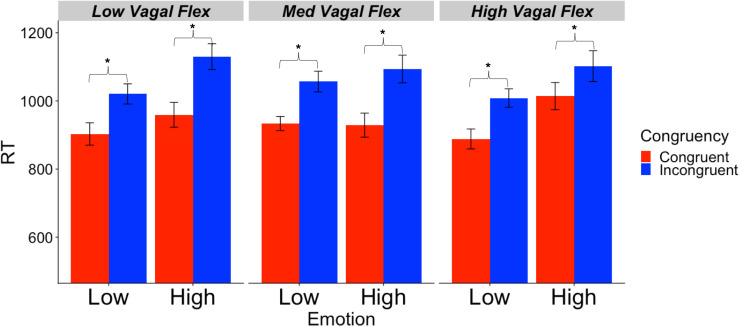
Mean RT (SE) as a function of vagal flexibility, emotion distractor condition, and Stroop congruency. Vagal flexibility is based on grouping of Reactivity_SD_ scores after a tertile split (low: *n* = 15, medium: *n* = 14, high: *n* = 15). *p < 0.01.

**FIGURE 3:**
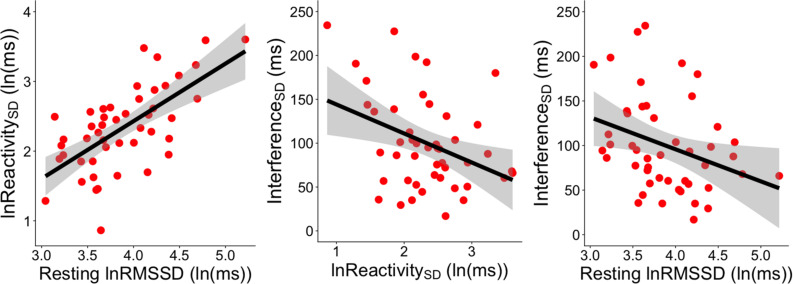
Significant correlations between RMSSD metrics and Interference_SD_. The **left panel** shows the correlation between resting lnRMSSD and the SD of RMSSD reactivity scores [Reactivity_SD_]. The **middle panel** shows the correlation between the SD of RMSSD reactivity scores [Reactivity_SD_] and the SD of RT interference scores across socioemotional conditions [Interference_SD_]. The **right panel** depicts the correlation between resting lnRMSSD and the SD of RT interference scores across socioemotional conditions [Interference_SD_]. RMSSD reactivity is defined as task RMSSD - BL RMSSD. RT interference is defined as incongruent RT - congruent RT. Each scatterpoint represents a single subject. Shaded regions indicate 95% confidence bands.

Descriptive statistics for RMSSD and performance metrics appear in Tables 1, 2. In the ANOVA examining Stroop RT, there was a significant main effect of congruency, such that RTs were longer during incongruent relative to congruent trials (for ANOVA results, see Table 3). Demonstrating a Stroop color–word interference effect, planned comparisons revealed that RTs were significantly longer for incongruent relative to congruent trials across all conditions (LELS: *t* = −5.75, *p* < 0.001; HELS: *t* = −7.46, *p* < 0.001, LEHS: *t* = −8.66, *p* < 0.001; HEHS: *t* = −7.18, *p* < 0.001) (MacLeod and MacDonald, 2000). There was also a significant vagal flexibility × congruency × emotion interaction. This interaction was probed by first examining simple two-way congruency × emotion interactions in each vagal flexibility group separately. This analysis revealed a congruency × emotion interaction at low, *F*(1, 45) = 7.49, *p* = 0.009, and medium flexibility, *F*(1, 45) = 4.21, *p* = 0.046, but not high flexibility, *F*(1, 45) = 2.71, *p* = 0.107, suggesting that congruency effects were significantly modulated by emotional distractors at low and medium but not high vagal flexibility. We next sought to clarify the congruency × emotion interactions in terms of the directional changes in congruency effects between emotion conditions. This was handled by testing pairwise comparisons of RT between congruency conditions (representing magnitude of interference effects) at different levels of emotion and flexibility, collapsing across the social factor (see Figure 2). At low flexibility (tertile 1), the interference effect (*t* test contrast of RT between congruent and incongruent trials) nearly doubled from low, *t*(15) = −4.67, *p* < 0.001, to high emotion, *t*(15) = −8.02, *p* < 0.0001. These *t* contrasts were significantly different between low and high emotion, as suggested by a significant two-way congruency × emotion interaction at low flexibility, *F*(1, 45) = 7.49, *p* = 0.009. The same pattern of findings was found at medium flexibility (tertile 2): low emotion *t*(14) = −3.91, *p* = 0.002, high emotion: *t*(14) = −6.54, *p* < 0.0001, where these contrasts were also significantly different, congruency × emotion: *F*(1, 45) = 4.21, *p* = 0.046. However, at high flexibility (tertile 3), the interference effects did not as heavily change from low emotion, *t*(15) = −5.99, *p* < 0.0001, to high emotion: *t*(15) = −4.26, *p* < 0.001. Consistently, these *t* contrasts were not significantly different between low and high emotion conditions, congruency × emotion, *F*(1, 45) = 2.71, *p* = 0.107. Taken together, these findings indicate significant emotion distraction effects (i.e., significant increases in interference from low to high emotional distractors) at low and medium but not high vagal flexibility. In other words, high vagal flexibility was associated with relatively weaker emotional distraction effects. Such results clarify the specific patterns of performance variability within the association between performance stability and vagal flexibility (reported in Figure 3). There was neither a significant main effect for social nor a significant vagal flexibility × congruency × social interaction (Table 3). These findings indicate no robust social distraction effects for any of the vagal flexibility groups.

**TABLE 1:** Means (SD) of primary study variables.

Age (years)	19.51 (1.46)
BMI (kg/m^2^)	24.10 (4.16)
Resting IBI (ms)	840.39 (131.58)
Resting HF-peak (Hz)	0.232 (0.060)
Resting RMSSD (ms)	54.79 (30.62)
Resting lnRMSSD (ln[ms])	3.88 (0.479)
HF-peak_MEAN_ (Hz)	−0.038 (0.051)
HF-peak_SD_ (Hz)	0.069 (0.041)
Reactivity_MEAN_ (ms)	1.21 (10.38)
Reactivity_SD_ (ms)	12.37 (8.15)
lnReactivity_SD_ (ln[ms])	2.33 (0.605)
Interference_MEAN_ (ms)	130.35 (78.38)
Interference_SD_ (ms)	100.00 (53.55)

*BMI, body mass index; IBI, interbeat interval; HF-peak, peak frequency in high-frequency (AR) spectral band of AR time-frequency analysis of IBI time series; RMSSD, root mean square of successive differences in interbeat intervals; HF-peak_MEAN_, mean of HF-peak reactivity scores; HF-peak_SD_, SD of HF-peak reactivity scores across socioemotional conditions; Reactivity_MEAN_, mean of RMSSD reactivity scores; Reactivity_SD_, SD of RMSSD reactivity scores; lnReactivity_SD_, natural logarithm of the SD of RMSSD reactivity scores; Interference_MEAN_, mean of Stroop interference scores; Interference_SD_, SD of Stroop interference scores.*

**TABLE 2:** Means (SD) of HRV and performance metrics by condition (*n* = 47).

	LELS		HELS		LEHS		HEHS	
	Baseline	Task	Baseline	Task	Baseline	Task	Baseline	Task
IBI (ms)	806.44 (119.48)	807.75 (122.13)	809.39 (114.19)	814.07 (120.12)	807.41 (115.05)	808.40 (111.84)	791.20 (155.12)	829.71 (125.26)
HF-peak (ms)	0.217 (0.062)	0.266 (0.077)	0.231 (0.068)	0.269 (0.078)	0.224 (0.073)	0.260 (0.078)	0.220 (0.065)	0.248 (0.080)
HF-peak reactivity (ms)	−0.049 (0.084)		−0.038 (0.099)		−0.036 (0.089)		−0.027 (0.088)	
RMSSD (ms)	50.37 (26.44)	48.72 (26.18)	49.76 (25.67)	47.81 (27.31)	48.67 (25.97)	45.28 (24.46)	46.33 (22.98)	48.47 (24.14)
RMSSD reactivity (ms)	1.66 (15.74)		1.95 (20.09)		3.39 (15.56)		-2.14 (13.74)	

	**LELS**		**HELS**		**LEHS**		**HEHS**	
	**Congruent**	**Incongruent**	**Congruent**	**Incongruent**	**Congruent**	**Incongruent**	**Congruent**	**Incongruent**

RT (ms)	916.83 (260.72)	1029.91 (268.32)	946.51 (277.42)	1080.38 (296.94)	898.79 (277.70)	1025.95 (287.67)	990.12 (317.13)	1137.39 (377.32)
Interference (ms)	113.09 (134.73)		133.87 (123.06)		127.15 (100.70)		147.27 (140.61)	
Accuracy (% correct)	98.54 (3.25)	97.47 (2.34)	98.27 (3.11)	96.72 (3.90)	98.14 (3.43)	96.19 (3.23)	98.27 (3.11)	96.76 (4.36)

*IBI, interbeat interval; HF-peak, peak frequency in high-frequency (AR) spectral band of AR time-frequency analysis of IBI time series; HF-peak reactivity, HF-peak reactivity (Task-BL); RMSSD reactivity, root mean square of successive differences [RMSSD] reactivity (Task-BL) scores; Interference, Incongruent RT – Congruent RT; Accuracy, % correct Stroop trials. LELS, Low Emotion; Low Social; LEHS, Low Emotion, High Social; LEHS, Low Emotion, High Social; HEHS, High Emotion, High Social.*

**TABLE 3:** Stroop RT by congruency, and distractor conditions (emotion, social).

Predictor	*SS*_Num_	*SS*_Den_	*F*	*p*	$\eta_{\text{G}}^{2}$
Flex	230203.79	22054131.26	0.47	0.497	0.007
Cong	1597074.81	539303.15	133.26	0.000	0.048
Emo	470270.86	2072747.14	10.21	0.003	0.014
Soc	36316.56	3058750.56	0.53	0.469	0.001
Flex × Cong	25870.29	539303.15	2.16	0.149	0.001
Flex × Emo	11573.33	2072747.14	0.25	0.619	0.0004
Flex × Soc	76679.23	3058750.56	1.13	0.294	0.002
Cong × Emo	9829.16	274213.69	1.61	0.211	0.0003
Cong × Soc	4431.09	299911.23	0.66	0.419	0.0001
Emo × Soc	88325.77	3441586.82	1.15	0.288	0.003
Flex × Cong × Emo	29261.93	274213.69	4.80	0.034	0.001
Flex × Cong × Soc	405.49	299911.23	0.06	0.806	0.00001
Flex × Emo × Soc	164940.69	3441586.82	2.16	0.149	0.005
Cong × Emo × Soc	2.66	283883.67	0.00	0.984	<0.0001
Flex × Cong × Emo × Soc	896.96	283883.67	0.14	0.708	<0.0001

*Flex, Vagal Flexibility Group; Cong, Congruency; Emo, Emotional; Soc, Social; SS_Num_, sum of squares numerator; SS_Den_, sum of squares denominator; $\eta_{G}^{2}$, generalized eta-squared.*

When testing socioemotional effects on lnRMSSD, there was a significant main effect of vagal flexibility. That is, the high flexibility group had higher average levels of lnRMSSD across conditions (baseline and tasks) compared to the low flexibility group, *t*(30) = −4.16, *p* < 0.001. There was no significant difference in lnRMSSD between the low and medium flexibility groups, *t*(29) = −1.32, *p* = 0.198, but the high flexibility group had higher lnRMSSD scores relative to the medium flexibility group, *t*(29) = −3.08, *p* = 0.005. There were no other significant main effects or interactions (*p* > 0.05, two-tailed) in the model (Table 4). Planned comparisons revealed that, across subjects, lnRMSSD did not significantly differ between baseline and task for any condition (LELS: *t* = 1.24, *p* = 0.221; HELS: *t* = 0.930, *p* = 0.357; LEHS: *t* = 1.49, *p* = 0.143; HEHS: *t* = −1.18, *p* = 0.242). Taken together, these results indicate no consistent directional RMSSD response evoked by the Stroop + distractor task at any level of vagal flexibility.

**TABLE 4:** LnRMSSD by task (baseline, task), and distractor conditions (emotion, social).

Predictor	*SS*_Num_	*SS*_Den_	*F*	*p*	$\eta_{\text{G}}^{2}$
Flex	22.04	52.49	18.89	0.000	0.246
Task	0.03	3.45	0.38	0.543	0.0004
Emo	0.02	2.14	0.46	0.502	0.0003
Soc	0.06	2.11	1.24	0.272	0.0009
Flex × Task	0.00	3.45	0.04	0.845	<0.0001
Flex × Emo	0.02	2.14	0.51	0.478	0.0004
Flex × Soc	0.13	2.11	2.86	0.097	0.002
Task × Emo	0.02	1.77	0.53	0.471	0.0003
Task × Soc	0.01	2.06	0.17	0.679	0.0001
Emo × Soc	0.10	1.50	3.15	0.083	0.002
Flex × Task × Emo	0.00	1.77	0.02	0.894	<0.0001
Flex × Task × Soc	0.00	2.06	0.00	0.951	<0.0001
Flex × Emo × Soc	0.07	1.50	2.02	0.162	0.001
Task × Emo × Soc	0.06	2.11	1.18	0.284	0.001
Flex × Task × Emo × Soc	0.13	2.11	2.82	0.100	0.002

*Flex, Vagal Flexibility Group; Task, Baseline versus Stroop task; Emo, Emotional; Soc, Social; SS_Num_, sum of squares numerator; SS_Den_, sum of squares denominator; $\eta_{G}^{2}$, generalized eta-squared.*

### Correlations Between Performance Metrics

In order to clarify the psychological significance of our performance stability metric, we analyzed its correlations with other performance metrics (accuracy and mean RT). There was a significant positive relation between Interference_SD_ and mean RT across all Stroop and socioemotional conditions (*r* = 0.463, *p* = 0.001, 95% confidence interval [CI] [0.203, 0.662]). Follow-up tests reveal that Interference_SD_ was related to each RT score separately (*p* < 0.05, results not presented). The correlation between Interference_SD_ and mean Stroop accuracy (proportion trials correct) scores across conditions was not significant. Similarly, Interference_SD_ was not significantly associated with accuracy scores when separated by condition (*p* > 0.05, results not presented).

### Correlations Between HRV and Performance Metrics

Resting lnRMSSD was positively correlated with Reactivity_SD_ (*r* = 0.654, *p* < 0.0001, 95% CI [0.452, 0.793]) but not with Reactivity_MEAN_ (*r* = 0.014, *p* = 0.925, 95% CI [-0.274, 0.300]). In other words, subjects with higher levels of resting lnRMSSD exhibited greater differences in RMSSD reactivity between the conditions (Figure 3).

Table 5 contains Pearson zero-order correlations testing associations between RMSSD and Stroop interference metrics, as well as analogous PCCs that controlled for sex, age, BMI, and HF-peak. LnReactivity_SD_ was significantly correlated with Interference_SD_ but not Interference_MEAN_. That is, larger differences in RMSSD reactivity between the socioemotional conditions were related to more stability in interference across the same conditions (Figure 3). Reactivity_MEAN_ was not significantly associated with Interference_MEAN_ or Interference_SD_. Similarly, there were no significant correlations when examining RMSSD reactivity and interference scores separated by condition (see Supplementary Results).

**TABLE 5:** Correlations (r) and partial correlation coefficients (PCC) between HRV [RMSSD] and interference metrics.

	Interference_MEAN_		Interference_SD_	
	*r* (*p*-value) [95% CI]	*PCC* (*p*-value) [95% CI]	*r* (*p*-value) [95% CI]	*PCC* (*p*-value) [95% CI]
lnReactivity_SD_	−0.131 (0.382) [−0.403, 0.163]	−0.084 (0.593) [−0.375, 0.222]	−0.374 (0.010) [−0.597, −0.097]*	−0.378 (0.012) [−0.609, −0.088]*
Reactivity_MEAN_	−0.113 (0.449) [−0.388, 0.180]	−0.143 (0.361) [−0.425, 0.165]	−0.075 (0.615) [−0.355, 0.217]	−0.068 (0.663) [−0.361, 0.237]
Resting lnRMSSD	−0.101 (0.501) [−0.377, 0.192]	−0.061 (0.700) [−0.354, 0.244]	−0.322 (0.027) [−0.557, −0.038]*	−0.322 (0.035) [−0.567, −0.024]*

lnReactivity_SD_, natural logarithm of the SD of RMSSD reactivity scores; Reactivity_MEAN_, mean of RMSSD reactivity scores; Resting lnRMSSD, natural logarithm of resting RMSSD; Interference_MEAN_, mean of Stroop interference scores; Interference_SD_, SD of Stroop interference scores. *p < 0.05 (two-tailed).

Like Reactivity_SD_, resting lnRMSSD was significantly related to Interference_SD_ but not to Interference_MEAN_. The relation between resting lnRMSSD and Interference_SD_ was negative in direction, indicating that higher levels of resting lnRMSSD were associated with relatively greater stability in Stroop interference across the socioemotional conditions (Figure 3). Clarifying its null relation with Interference_MEAN_, resting lnRMSSD was not significantly associated with any of the interference scores separated by condition (see Supplementary Results). Taken together, results indicate that resting vagal activity and vagal flexibility were both related to the degree of stability in (but not mean levels of) inhibition performance.

### Mediational Findings

**FIGURE 4:**
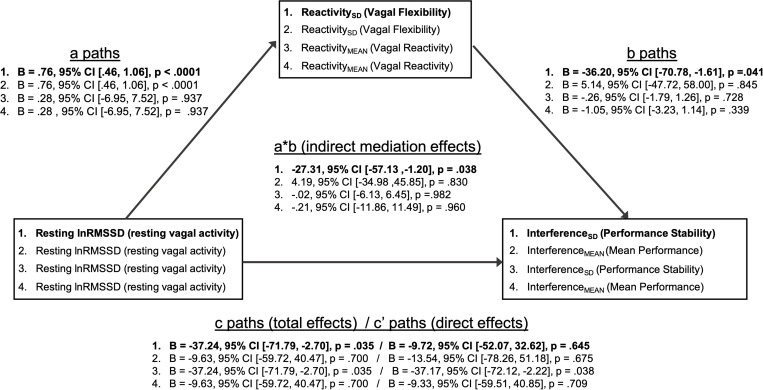
Summary of mediation models. Mediation models (1–4) test whether vagal flexibility and mean vagal reactivity mediated relations between resting vagal activity and performance metrics. The indirect effects (path a * path b) tested mediation. Two-tailed *p*-values and 95% confidence intervals for the indirect effects were computed using an approximate Bayesian Monte Carlo method (# simulations = 10,000). Statistics for path coefficients include unstandardized regression coefficients (*B*), two-tailed *p*-values, and 95% confidence intervals. Path a represents the relations of resting vagal activity to vagal flexibility and mean vagal reactivity. Path b represents the relations of vagal flexibility and mean vagal reactivity to performance stability and mean performance. Path c represents the total effect of resting vagal activity on performance metrics before accounting for mediator (vagal flexibility or vagal reactivity). Path c’ represents the direct effect of resting vagal activity on performance metrics after adjusting for mediator. Bolded text indicates that the indirect/mediational effect for that model was statistically significant (*p* < 0.05). There was a significant indirect effect of resting vagal activity on performance stability through vagal flexibility (mediator). All other indirect effects were not statistically significant (*p* > 0.05).

Mediation models are summarized in Figure 4. The patterns of significant mediational relations (paths a, b, c) are identical to the correlations among vagal and performance metrics described above (Table 5). As hypothesized, there was a significant indirect effect (a ^∗^ b; mediational effect) of resting lnRMSSD on Interference_SD_ through Reactivity_SD_. Within this pathway, higher resting lnRMSSD was associated with higher Reactivity_SD_, and higher Reactivity_SD_ was related to lower Interference_SD_. The direct effect of resting lnRMSSD on interference_SD_ was not statistically significant, suggesting that vagal flexibility fully mediated the relation between resting vagal activity and inhibition performance stability.

In contrast, Reactivity_SD_ did not mediate the relation between resting lnRMSSD and Interference_MEAN_. Consistent with its null correlations with performance above (Table 5), Reactivity_MEAN_ did not significantly mediate associations between resting lnRMSSD and either performance metric (Interference_MEAN_ or Interference_SD_).

## Discussion

The current study is the first to show that vagal flexibility in response to varying challenges mediates the relation between resting vagal activity and stability in cognitive performance across the same challenges. Although the present results require independent replication, our main findings supported our hypotheses and were as follows: (1) vagal flexibility fully mediated the link between resting vagal activity and stability in cognitive inhibition performance. (2) Within this mediational pathway, individuals with higher resting vagal activity had greater modulation of vagal responses to varying socioemotional distractors. (3) This higher vagal flexibility was in turn linked to greater stability in inhibition performance across the varying distractors. Results also indicate that high vagal flexibility was related to weaker emotional distraction effects. Here, vagal flexibility may have promoted performance stability by suppressing the deleterious effects of emotional distractors on inhibition performance. Contrary to our hypothesis, vagal flexibility did not significantly mediate a relationship between resting vagal activity and mean inhibition performance (the latter relation was not statistically significant).

These findings are consistent with the theorized role of vagal function in the adaptation of top-down regulation to fit changing perturbations – a capacity that is thought be indexed better by IIV than by mean-based performance metrics (Thayer and Lane, 2000; Koffer and Ram, 2015). Similarly, unlike vagal flexibility (estimated as IIV in vagal reactivity), metrics reflecting the degree of vagal reactivity were not significantly associated with resting HRV or any performance metric. These results suggest that prior studies’ attempts at operationalizing vagal flexibility as mean vagal reactivity could be missing important within-person dynamics in vagal reactivity (e.g., Muhtadie et al., 2015; Human and Mendes, 2018).

The findings mentioned are among the first to empirically support the theorized role of vagal flexibility in adaptive cognitive inhibition. In these perspectives, individuals with higher resting vagal activity are believed to exhibit adaptive cognition/behavior during challenges, because they can dynamically regulate vagal outflow to fit the self-regulatory requirements of the situation (Porges, 2007; Thayer and Lane, 2009). Although the findings are not necessarily surprising in the light of vagal theory, they bridge this theory with rigorous empirical testing. Such empirical validation is a prerequisite for substantiating robust mechanisms that can be leveraged in applied clinical/operational domains. Although preliminary, our findings support vagal flexibility as a potential state-based mechanism by which vagal activity at rest (a ubiquitous individual difference metric) contributes to cognition during stress.

It should be mentioned that, like the previous study (Spangler et al., 2018a), HRV metrics in the current study were associated with stability in cognitive inhibition (e.g., Stroop interference), specifically. The specificity of findings to inhibition is consistent with prior work that has strongly linked vagal activity to inhibitory control (i.e., Stroop interference, down-regulation of negative emotion). Indeed, multiple perspectives posit cardiac vagal activity as an indicator of inhibitory processes spanning skeletal motor, visceral, and cognitive systems (Jennings, 1992; Thayer and Lane, 2009).

### Importance of Dynamic IIV Metrics

As predicted, mediational relations were stronger when analyzing dynamic measures of IIV relative to static metrics of vagal activity and performance (i.e., mean-based metrics; vagal reactivity and performance scores for each condition). Stability in inhibition performance was related to greater intertask changes in vagal reactivity (vagal flexibility) but not to traditional estimates reflecting the degree of vagal reactivity (e.g., Porges et al., 1996; Mathewson et al., 2010; Muhtadie et al., 2015). Contrary to our hypotheses, resting vagal activity and vagal flexibility were not related to mean inhibition performance (i.e., Stroop interference), and similarly, vagal flexibility did not mediate the relation between resting vagal activity and mean performance. This lack of correlation between vagal metrics and general levels of inhibition performance is contrary to previous studies (e.g., Johnsen et al., 2003; Hovland et al., 2012). Yet, the correlation between HRV and mean-based executive function metrics have been shown to be small in size and unstable across studies (Mann et al., 2015; Laborde and Mosley, 2016; Zahn et al., 2016a, b; Holzman and Bridgett, 2017). Taken together with present results, this raises the possibility that – relative to mean-based metrics – IIV metrics of HRV/performance might better index the dynamic frontal lobe and inhibitory control functions theoretically linked to vagal activity (Kelly et al., 2007; MacDonald et al., 2009; Lane et al., 2013). It should be noted that a larger sample size could have increased our ability to detect significant associations involving static and mean-based, metrics of performance and HRV. Present results therefore require independent replication.

Nevertheless, the utility of dynamic relative to static/mean-based metrics is corroborated by prior studies from our research group. Speaking to the importance of IIV in vagal responses, we recently reported that individual differences in vagal flexibility (changes in vagal activity between tasks) but not vagal reactivity (Task-Baseline) were related to stability in inhibition performance across low and high stress (Spangler et al., 2018a). Emphasizing the importance of IIV in performance, the same study also reported that vagal flexibility was associated with stability of inhibition performance but not the degree of inhibition performance (i.e., mean inhibition, condition-level inhibition scores). We found similar findings in a separate study using consistency, a different IIV performance metric (Spangler et al., 2018b). Specifically, resting vagal activity was related to RT consistency but not to mean RT. While performance stability (the metric used in the current study) indexes exogenous task-related changes in performance, consistency reflects the degree of trial-to-trial changes in performance likely due to endogenous influences (Christensen et al., 1999; Hultsch et al., 2002). Although different, stability and consistency are both inversely linked to neurocognitive constructs including frontal lobe function and integrity of frontal-subcortical pathways (Stuss et al., 2003; Jones et al., 2018). Taken together with our current findings, it is possible that HRV’s stronger relations to stability and consistency (relative to mean performance) are due to HRV tapping into a general IIV mechanism (Hultsch et al., 2002; MacDonald et al., 2006; MacDonald et al., 2009). Future work should examine if these IIV metrics are differentially related to HRV, in order to clarify the neurocognitive mechanisms of vagal function (Li et al., 2004).

### Vagal Activity, Adaptive Responding, and Potential Mechanisms

Some situations call for cognitive flexibility and others for cognitive stability. The adaptive organism is able to adopt a pattern of stability or flexibility that best fits the situation (Miller and Cohen, 2001; Armbruster et al., 2012). Expanding on the prior study (Spangler et al., 2018a), present findings are consistent with vagal activity supporting patterns of cognition (flexibility vs. stability) that are most appropriate or adaptive for the situation (Thayer and Lane, 2009; Thayer et al., 2012). In the prior study (Spangler et al., 2018a), higher vagal flexibility was associated with less stability in response inhibition performance, which we interpreted to be an adaptive response. In that study, decreased inhibition over motor behavior during proximal physical threat (i.e., pain) is functional for releasing restraint on coping responses that are critical for survival (Nesse, 2005). In the present study, higher vagal flexibility instead related to greater stability in inhibition performance amid stimuli that do not pose immediate physical threat. In this particular context, greater stability is the adaptive response. Because changes in cognition due to mild motivational stimuli confer less survival value, those stimuli instead act as salient distractors whose influence must be suppressed to maintain stable performance (Dolcos and McCarthy, 2006). Present findings imply that individuals with high vagal flexibility had greater performance stability because they potentially suppressed the deleterious effects of emotional (but not social) distractors on performance. This notion is supported by present ANOVA findings. Specifically, compared to those with high vagal flexibility, individuals with low vagal flexibility had stronger decreases in inhibition performance from low to high emotional distractors – i.e., stronger patterns of emotional distraction (Dolcos and McCarthy, 2006; Most et al., 2007; West et al., 2009; Hodsoll et al., 2011). Across all level of vagal flexibility, social auditory stimuli had no significant distraction effects on performance.

Relations of vagal flexibility to performance stability and emotional distraction are consistent with extant theory that links vagal activity to the PFC-mediated regulation of attention, emotion, and cardiovascular arousal (Thayer et al., 2012; Lane et al., 2013; Park and Thayer, 2014). Indeed, higher performance stability has been associated with superior structural and functional integrity of the frontal lobes and their interconnections with subcortical “arousal” structures (Stuss et al., 2003; Jones et al., 2018). Similarly, the suppression of emotional distraction effects has been correlated with PFC activity in response to motivational stimuli (e.g., emotional intensity of stimuli; Dolcos and McCarthy, 2006; Weissman et al., 2006; Lane et al., 2009, 2013; Thayer and Lane, 2009; Armbruster et al., 2012; Silvers et al., 2014). In the light of previous work, high vagal flexibility in the current study may therefore reflect changes in the degree to which attention (and/or arousal) to emotional distractors is optimally regulated by the PFC. Such proposed mechanisms require validation in future studies. Nevertheless, we conceptualize performance stability in the current study as being *adaptive* for two reasons. First, the prior literature reports greater emotional distraction and lower performance stability among individuals with neurocognitive dysfunction and/or psychopathology (Krause-Utz et al., 2012; Bangen et al., 2019). Second, we found that higher performance stability in the current study was related to faster RT, a pattern of performance linked to adaptive neurocognitive function (Dixon et al., 2007; Mella et al., 2016).

Speaking further to neural mechanisms, the notion that vagal flexibility mediates the relation between resting vagal activity and performance might be clarified with a resource account of these metrics’ neural correlates (Thayer and Lane, 2009). Vagal flexibility in the current study may have represented the state-related activation of ample PFC resources whose reserves are reflected by tonic vagal activity at rest. According to the neurovisceral integration model, tonic vagal activity is posited to index the amount of PFC resources (i.e., PFC capacity) that can be utilized for emotional/cognitive regulation (Thayer and Lane, 2009; Park et al., 2014; Laborde et al., 2018). Task-related shifts in vagal activity are believed to index the active deployment of such PFC resources for discrete regulatory efforts, including attentional regulation (Ingjaldsson et al., 2003; Butler et al., 2006; Segerstrom and Nes, 2007; Park and Thayer, 2014). Supporting those notions, Lane et al. (2013) reported that within-person changes in HRV were accompanied by shifts in ventromedial PFC activity across changing demands on an affective set-shifting task. Critically, these adaptive shifts in PFC activity were stronger in individuals with higher resting HRV, perhaps because these individuals had more ample PFC resources for regulating emotion (Smith et al., 2016). Such active shifts in PFC responses are thought to be critical for cognitive stability vis-à-vis regulating attention to changing motivational distractors (Dolcos and McCarthy, 2006; Weissman et al., 2006; Armbruster et al., 2012). Taken together, tonic vagal activity and vagal flexibility in the current study may represent a capacity and adaptive utilization of PFC resources, respectively, which are both critical for suppressing emotional distraction. Future work should examine the neural correlates of individual differences in vagal flexibility to directly test such inferences.

### Implications

As noted earlier, present findings are among the first to support aspects of polyvagal theory and neurovisceral integration that highlight the situationally appropriate titration of vagal responses (Porges et al., 1996; Thayer and Lane, 2000). The present work revitalizes notions of dynamic variability, ecological diversity, and organismic flexibility, which permeated these theories as they were presented decades ago (Porges, 1995a, b; Friedman, 2007; Thayer and Lane, 2000). Of course, present findings are unable to shed light on all major elements of polyvagal and neurovisceral integration theories, and each of these theories is not without its critics. In polyvagal theory, the functional differentiation of two vagal fiber types in humans is not well supported (Farmer et al., 2016), and its claims about the specificity of myelinated vagal fibers to mammals has been challenged (Monteiro et al., 2018). In neurovisceral integration, evidence for common neural structures that regulate both cognition and vagal activity are mixed (Thayer et al., 2012; Jennings et al., 2015), and some studies have reported that the links of HRV to cognition (Zahn et al., 2016a) and well-being (Sloan et al., 2017) are weak in effect size (for deeper discussion of contextual issues impacting such effects, see Laborde and Mosley, 2016; Zahn et al., 2016b). While the latter criticisms are important for theory refinement, falsification of some elements of a theory does not invalidate the theory in its entirety, leaving key questions about vagal flexibility open for continued study.

Static measures of resting HRV are thought to characterize trait vagal function as it relates to cognition. Expanding on this notion, present findings suggest that trait vagal function should also be understood in terms of intraindividual dynamics across numerous situations. As is noted above in our PFC resource account, one important aspect of trait vagal function reflects self-regulatory capacity, which can be presumably measured when the individual is not taxed and maximal resources are preserved (i.e., during resting state). However, according to the landmark work by Mischel and Shoda (1995), traits can be conceptualized by considering context and going beyond static metrics (i.e., single-condition or mean-based metrics). Traits in this perspective are better understood as characteristic patterns of IIV across multiple situations. Through this lens, individual differences in vagal flexibility may represent the situational dynamics of trait vagal function, thus serving as the mechanism of resting HRV’s effects on cognition. Indeed, significant between-subject differences in IIV of vagal activity (across multiple situations) have been reported, but the psychological significance of this complex variance structure has been largely ignored until now (Bertsch et al., 2012).

In the clinical domain, vagal activity has increasingly been linked to anxiety and trauma-based disorders (Friedman, 2007; Tan et al., 2013). These disorders are typically characterized by difficulties in emotion regulation and deficits in efficient attentional allocation. Although a strategy such as response inhibition can be important for emotion regulation, consistency in efficient situational responding usually does not rely on a single regulatory strategy; both the presence of multiple strategies and the ability to access strategies that are most metabolically economical for the situation are ideal for responding (Bonanno and Burton, 2013). Research at both the bench and bedside could benefit from better understanding the relations between vagal flexibility and the ability to select the appropriate regulatory strategy (i.e., regulatory flexibility).

### Limitations and Future Directions

Limitations of the present study include our inability to directly measure the regulatory processes that drive individual differences in vagal flexibility. Nevertheless, the dual-task paradigm in tandem with prior theory helps bolster the involvement of PFC-mediated regulation. In addition, we did not directly control for respiration or other physiological variables. Future studies should examine variability in autonomic responses with a greater diversity of autonomic measures such as pre-ejection period and skin conductance. Related to this point, sympathetic contributions to physiological flexibility may reveal important aspects of cognitive/behavioral adaptability (Wright and Kirby, 2001). Although the focus of the current study was on the relation between vagal activity and cognitive inhibition, researchers should assess other facets of cognition with different performance tasks (e.g., working memory, set shifting, planning, distractor inhibition). Another limitation of the present study is its small sample size, making present mediational and ANOVA findings provisional upon independent replication. The small sample size also limited the ability to model more complex patterns of variability beyond what was analyzed in the current study. Future studies could utilize multilevel regression or structural equation modeling, for example, to more richly characterize IIV based on patterns of directional intraindividual change in HRV/performance. Such an approach would clarify the interestingly complex patterns of variability in the data, including the present finding, where individuals with high vagal flexibility showed variable patterns of directional HRV change across conditions. Relatedly, the present study is limited in characterizing the rich IIV in vagal activity within tasks, a form of dynamic vagal change that has been examined mainly in children (e.g., Brooker and Buss, 2010). Given that these dynamic HRV metrics reflect within-task variability, they indicate modulation of vagal function in response to endogenous factors. However, the present study focused on variability in HRV (vagal flexibility) in response to exogenous demands that are of central focus to extant vagal theories (e.g., Thayer and Lane, 2000; Porges, 2007). Future work should examine *both* endogenously and exogenously driven vagal responses within the same paradigm to understand their roles in self-regulation. As aforementioned, current findings imply a potential role of vagal flexibility in trait vagal function. Current findings nevertheless do not speak to whether observed individual differences in vagal flexibility are stable and thus trait-like. Longitudinal studies are needed to address its test–retest reliability and, more importantly, the complex multi-timescale variance in vagal-performance relations. Lastly, the current sample was limited to college students, thus necessitating future studies to indicate whether findings generalize to other populations. Despite limitations, the present study is an important bridge between theory and empirical evidence regarding the role of vagal flexibility in adaptive cognition.

## Data Availability Statement

The datasets generated for this study are available on request to the corresponding author.

## Ethics Statement

The studies involving human participants were reviewed and approved by the Towson University Institutional Review Board. The patients/participants provided their written informed consent to participate in this study.

## Author Contributions

DS and JM wrote the manuscript collaboratively. JM executed the collection and analysis of physiological and behavioral metrics. DS executed the statistical analysis and inferences. Both authors contributed to the article and approved the submitted version.

## Conflict of Interest

The authors declare that the research was conducted in the absence of any commercial or financial relationships that could be construed as a potential conflict of interest.

## Footnotes

1 In the remainder of the article, we use the term HRV to specifically refer to vagally mediated HRV, which uniquely indexes cardiac vagal activity (Saul, 1990).

2 See Supplementary Appendix B for a glossary of potentially unfamiliar terms that are used in this article.

3 In the prior literature, some authors refer to IIV in performance across tasks as dispersion. However, we use “performance stability” because it is a more intuitive term for a broader audience.
