# Supplementary material for: Vagal Flexibility Mediates the Association Between Resting Vagal Activity and Cognitive Performance Stability Across Varying Socioemotional Demands
Source: Front Psychol. 2020 Sep 9;11:2093. doi: 10.3389/fpsyg.2020.02093 (PMC7509204; doi:10.3389/fpsyg.2020.02093)
Supplement: Supplementary file 1 [file Data_Sheet_1.docx]

**Supplemental Materials**

**Task and Vagal Flexibility Effects on Accuracy**

Although Stroop interference is most often indexed with Congruency effects on RT (MacLeod, 1991), we also examined Congruency effects on Stroop accuracy (proportion of correct responses) and the extent to which these effects varied by distractor condition and Vagal Flexibility group. These effects were tested with an ANOVA (see Supplementary Table S1). The only significant factor was Congruency. Planned comparisons revealed that accuracy was significantly decreased during incongruent relative to congruent trials for each socioemotional condition (LEHS: *t* = 3.43, *p* = .001; HELS: *t* = 2.22, *p* = .031; HEHS: *t* = 2.17, *p* = .035) except Low Emotion-Low Social where the effect was in the same direction but only trending toward statistical significance, *t* = 1.88, *p* = .067. There were no other significant main effects or interactions. These results suggest that Stroop accuracy (and Congruency effects on accuracy) did not differ between the socioemotional distractor conditions or the Vagal Flexibility groups.

**Supplementary Table 1.** Stroop Accuracy (Proportion of Correct Responses) by Congruency, and Distractor Conditions (Emotion, Social)

|  |  | |  | | |  | | |  | | | |  | |  | |  |  |
| --- | --- | --- | --- | --- | --- | --- | --- | --- | --- | --- | --- | --- | --- | --- | --- | --- | --- | --- |
| **Predictor** | ***SS_Num_*** | | ***SS_Den_*** | | | ***F*** | | | ***p*** | | | | **η^2^_G_** | |  | |  |  |
| Flex | | 39.61 | | 1444.49 | | | 0.60 | | | 0.551 | | 0.010 | |  |  |  |  |  |
| Cong | | 216.66 | | 368.43 | | | 25.87 | | | <.0001 | | 0.051 | |  |  |  |  |  |
| Emo | | 0.57 | | 366.58 | | | 0.07 | | | 0.796 | | 0.0001 | |  |  |  |  |  |
| Soc | | 15.8 | | 295.81 | | | 2.35 | | | 0.132 | | 0.004 | |  |  |  |  |  |
| Flex x Cong | | 26.35 | | 368.43 | | | 1.57 | | | 0.219 | | 0.006 | |  |  |  |  |  |
| Flex x Emo | | 9.91 | | 366.58 | | | 0.59 | | | 0.556 | | 0.002 | |  |  |  |  |  |
| Flex x Soc | | 0.35 | | 295.81 | | | 0.026 | | | 0.975 | | <.0001 | |  |  |  |  |  |
| Cong x Emo | | 0.01 | | 332.62 | | | 0.0015 | | | 0.969 | | <.0001 | |  |  |  |  |  |
| Cong x Soc | | 4.17 | | 396.29 | | | 0.46 | | | 0.500 | | 0.001 | |  |  |  |  |  |
| Emo x Soc | | 17.56 | | 301.5 | | | 2.56 | | | 0.1172 | | 0.004 | |  |  |  |  |  |
| Flex x Cong x Emo | | 31.41 | | 332.62 | | | 2.08 | | | 0.137 | | 0.008 | |  |  |  |  |  |
| Flex x Cong x Soc | | 15.67 | | 396.29 | | | 0.87 | | | 0.426 | | 0.004 | |  |  |  |  |  |
| Flex x Emo x Soc | | 23.28 | | 301.50 | | | 1.70 | | | 0.195 | | 0.006 | |  |  |  |  |  |
| Cong x Emo x Soc | | 5.09 | | 540.87 | | | 0.41 | | | 0.523 | | 0.001 | |  |  |  |  |  |
| Flex x Cong x Emo x Soc | | 30.75 | | 540.87 | | | 1.25 | | | 0.296 | | 0.008 | |  |  |  |  |  |
|  |  | |  | |  | | |  | | |  | | | | |  | |  |

*Note.* Flex = Vagal Flexibility Group, Cong = Congruency, Emo = Emotional, Soc = Social, *SS_Num_* = sum of squares numerator, *SS_Den_* =sum of squares denominator, η^2^_G_ = generalized eta-squared.

**Correlations Between RMSSD and RT Interference Metrics**

In order to clarify our primary findings, we also tested relations involving condition-level metrics for both RMSSD and RT interference. See Supplementary Table S2 for correlations between interference scores (separated by socioemotional condition) and primary RMSSD metrics. Supplementary Table S3 contains the correlations between RMSSD reactivity scores (separated by condition) and primary interference metrics. None of the correlations were statistically significant (two-tailed, p > .05). We therefore did not additionally control for covariates when examining these associations.

**Supplementary Table S2.** Zero-order Pearson correlations (p-values) [95% CI] between RMSSD metrics and Stroop RT interference scores separated by condition.

|  | | | | |
| --- | --- | --- | --- | --- |
|  | **LELS Interference** | **HELS Interference** | **LEHS Interference** | **HEHS interference** |
| **lnReactivity_SD_** | .109 (.467) [-.184, .384] | -.277 (.059) [-.523, .011] | .059 (.696) [-.233, .340] | -0.195 (.190) [-.456, .098] |
|  |  |  |  |  |
| **Reactivity_MEAN_** | .014 (.924) [-.274, .300] | -.050 (.740) [-.332, .241] | -.175 (.238) [-.440, .118] | -.097 (.517) [-.374, .196] |
|  |  |  |  |  |
| **Resting lnRMSSD** | .160 (.282) [-.133, .428] | -.122 (.415)  [-.395, .171] | -.036 (.810) [-.320, .254] | -.246 (.096) [-.498, .045] |
|  |  |  |  |  |
| **Reactivity**  **(by respective condition)** | .0002 (.999) [-.287, .287] | -.028 (.851) [-.313, .261] | -.107 (.475) [-.382, .186] | .018 (.903) [-.270, .304] |
|  | | | | |

lnReactivity_SD_ = natural logarithm of the SD of RMSSD reactivity scores, Reactivity_MEAN_ = mean of RMSSD reactivity scores, Resting lnRMSSD = natural logarithm of resting RMSSD. Reactivity (by respective condition) refers to the RMSSD reactivity score for the same condition as the corresponding interference score (e.g., LELS reactivity for LESL interference).

No significant correlations (p > .05, two-tailed)

**Supplementary Table S3.** Zero-order Pearson correlations (p-values) [95% CI] between RMSSD reactivity scores separated by condition and Stroop RT interference metrics

|  | | |
| --- | --- | --- |
|  | **Interference_MEAN_** | **Interference_SD_** |
| **LELS reactivity** | -.044 (.768) [-.327, .246] | .031 (.836) [-.258, .315] |
| **HELS reactivity** | -.057 (.704) [-.339, .234] | -.057 (.704) [-.339, .234] |
| **LEHS reactivity** | -.190 (.201) [-.452, .103] | -.193 (.193) [-.455, .100] |
| **HEHS reactivity** | .007 (.963) [-.281, .293] | .039 (.796) [-.251, .322] |
|  |  |  |

Interference_MEAN_ = mean of Stroop RT interference scores, Interference_SD_ = SD of Stroop RT interference scores.

No significant correlations (p > .05, two-tailed)

**Correlations Between RMSSD and Accuracy-based Interference Metrics**

For completeness, we also tested correlations between primary RMSSD metrics and Stroop accuracy interference metrics. Such accuracy metrics were computed in the same manner as Interference_MEAN_ and Interference_SD_ (see Data Reduction) except accuracy rates (proportion of correct responses) was utilized instead of RT. Accuracy-Interference_MEAN_ and Accuracy-Interference_SD_ indexed the average of and intra-individual variability in Stroop accuracy scores, respectively. As indicated in Supplementary Tables S4-S6, there were no statistically significant correlations between any of the RMSSD and accuracy-based interference measures. Consistent with these null correlations, there were no significant indirect mediational effects with Accuracy-Interference_MEAN_ or Accuracy-Interference_SD_ as dependent measures (see Supplemental Table S7).

**Supplemental Table S4**. *Correlations (r) and Partial Correlation Coefficients (PCC) between HRV [RMSSD] and Accuracy Interference Metrics*

|  |  | |  | |
| --- | --- | --- | --- | --- |
|  | **Accuracy-Interference_MEAN_** | | **Accuracy-Interference_SD_** | |
|  | ***r*** (*p*-value) [95% CI] | ***PCC*** (*p*-value) [95% CI] | ***r*** (*p*-value) [95% CI] | ***PCC*** (*p*-value) [95% CI] |
| **lnReactivity_SD_** | .147 (.323) [-.146, .417] | .195 (.210) [-.112, .468] | -.130 (.384) [-.402, .163] | -.115 (.464) [-.401, .192] |
| **Reactivity_MEAN_** | .011 (.940) [-.277, .298] | .043 (.783) [-.260, .339] | -.222 (.134) [-.479, .070] | -.229 (.139) [-.495, .077] |
| **Resting lnRMSSD** | -.040 (.790) [-.323, .250] | .009 (.953) [-.292, .309] | -.154 (.300) [-.423, .139] | -.136 (.383) [-.420, .171] |
|  |  |  |  |  |

lnReactivity_SD_ = natural logarithm of the SD of RMSSD reactivity scores, Reactivity_MEAN_ = mean of RMSSD reactivity scores, Resting lnRMSSD = natural logarithm of resting RMSSD, Interference_MEAN_ = mean of Stroop accuracy interference scores, Interference_SD_ = SD of Stroop accuracy interference scores. Accuracy was defined as the proportion of correct responses out of total responses. Stroop accuracy interference was computed with the following difference score for each socioemotional condition: Incongruent accuracy – Congruent accuracy.

*p < .05 (two-tailed)

**Supplementary Table S5.** Zero-order Pearson correlations (p-values) [95% CI] between RMSSD metrics and Stroop accuracy interference scores separated by condition.

|  | | | | |  |
| --- | --- | --- | --- | --- | --- |
|  | **LELS Interference** | **HELS Interference** | **LEHS Interference** | **HEHS interference** | |
| **lnReactivity_SD_** | -.029 (.849) [-.313, .261] | .256 (.083)  [-.034 .506] | -.180 (.226) [-.444, .113] | .170 (.253)  [-.123, .436] | |
|  |  |  |  |  | |
| **Reactivity_MEAN_** | -.156 (.296) [-.424, .138] | .032 (.830)  [-.257, .316] | .038 (.799) [-.252, .322] | .083 (.578)  [-.209, .362] | |
|  |  |  |  |  | |
| **Resting lnRMSSD** | -.053 (.725) [-.335, .238] | .057 (.706)  [-.234, .339] | -.230 (.119) [-.486, .061] | .106 (.480)  [-.187, .381] | |
|  |  |  |  |  | |
| **Reactivity**  **(by respective condition)** | .018 (.907) [-.271, .303] | .229 (.121)  [-.062, .485] | .003 (.986) [-.285, .290] | .001 (.994) [-.286, .288] | |
|  | | | | |  |

lnReactivity_SD_ = natural logarithm of the SD of RMSSD reactivity scores, Reactivity_MEAN_ = mean of RMSSD reactivity scores, Resting lnRMSSD = natural logarithm of resting RMSSD. Reactivity (by respective condition) refers to the RMSSD reactivity score for the same condition as the corresponding interference score (e.g., LELS reactivity for LESL interference). Accuracy was defined as the proportion of correct responses out of total responses. Stroop accuracy interference was computed with the following difference score for each socioemotional condition: Incongruent accuracy – Congruent accuracy.

No significant correlations (p > .05, two-tailed)

**Supplementary Table S6.** Zero-order Pearson correlations (p-values) [95% CI] between RMSSD reactivity scores separated by condition and Stroop accuracy interference metrics

|  | | |
| --- | --- | --- |
|  | **Accuracy-Interference_MEAN_** | **Accuracy-Interference_SD_** |
| **LELS reactivity** | -.010 (.946) [-.296, .278] | -.166 (.264) [-.433, .127] |
| **HELS reactivity** | .226 (.126) [-.065, .482] | .040 (.791) [-.250, .323] |
| **LEHS reactivity** | -.152 (.306) [-.421, .141] | -.236 (.110) [-.490, .054] |
| **HEHS reactivity** | -.112 (.453) [-.387, .181] | -.270 (.066) [-.517, .018] |
|  |  |  |

Accuracy-Interference_MEAN_ = mean of Stroop accuracy interference scores, Accuracy-Interference_SD_ = SD of Stroop accuracy scores.

Accuracy was defined as the proportion of correct responses out of total responses. Stroop accuracy interference was computed with the following difference score for each condition: Incongruent accuracy – Congruent accuracy.

No significant correlations (p > .05, two-tailed)

**Supplementary Table S7.** Indirect mediational effects with accuracy-based interference

metrics as dependent measures

|  | **Indirect effect: B (p-value) [95% CI]** |
| --- | --- |
| **Resting lnRMSSD 🡪 Reactivity_SD_ 🡪 Acc-Interference_SD_** | -.172 (.800) [-1.58, 1.20] |
| **Resting lnRMSSD 🡪 Reactivity_SD_ 🡪 Acc-Interference_MEAN_** | .878 (.110) [-.211, 2.13] |
| **Resting lnRMSSD 🡪 Reactivity_MEAN_🡪 Acc-Interference_SD_** | -.014 (.940) [-.481, .430] |
| **Resting lnRMSSD 🡪 Reactivity_MEAN_ 🡪 Acc-Interference_MEAN_** | .006 (.999) [-.233, .250] |
|  |  |

Resting lnRMSSD = natural logarithm of resting RMSSD lnReactivity_SD_ = natural logarithm of the SD of RMSSD reactivity scores, Reactivity_MEAN_ = mean of RMSSD reactivity scores, Accuracy-Interference_MEAN_ = mean of Stroop accuracy interference scores, Accuracy-Interference_SD_ = SD of Stroop accuracy scores.

Accuracy was defined as the proportion of correct responses out of total responses. Stroop accuracy interference was computed with the following difference score for each condition: Incongruent accuracy – Congruent accuracy.

There were no significant indirect effects (p > .05, two-tailed)
