# Supplementary material for: Vagal Flexibility Mediates the Association Between Resting Vagal Activity and Cognitive Performance Stability Across Varying Socioemotional Demands
Source: Front Psychol. 2020 Sep 9;11:2093. doi: 10.3389/fpsyg.2020.02093 (PMC7509204; doi:10.3389/fpsyg.2020.02093)
Supplement: Supplementary file 2 [file Data_Sheet_2.docx]

Appendix A

Characteristics for IADS sounds selected for socioemotional tasks

| **Condition** | **IADS No.** | **Description** | **Pleasure (Mean, SD)** | **Arousal (Mean, SD)** |
| --- | --- | --- | --- | --- |
|  | 255 | Person groaning & vomiting | 2.08 (1.78) | 6.59 (2.08) |
|  | 260 | Several babies crying | 2.04 (1.39) | 6.87 (2.13) |
|  | 261 | Single baby crying | 2.75 (1.68) | 6.51 (1.96) |
| HEHS | 275 | Frightening screams | 2.05 (1.62) | 8.16 (2.15) |
|  | 277 | Frightened screaming | 1.63 (1.13) | 7.79 (1.63) |
|  | 279 | Screaming & attacking | 1.68 (1.31) | 7.95 (2.22) |
|  | 285 | Screaming & attacking | 1.80 (1.56) | 7.79 (2.01) |
|  | 286 | Screaming & gunshot | 1.68 (1.18) | 7.88 (1.72) |
|  | **Average ratings** | | **1.96 (1.46)** | **7.44 (1.99)** |
|  | 116 | Bees buzzing loudly | 3.02 (1.65) | 6.51 (2.13) |
|  | 422 | Car tires skidding | 1.86 (1.27) | 7.88 (1.38) |
|  | 424 | Tires skidding & car crash | 1.57 (0.90) | 8.21 (1.35) |
|  | 624 | Air raid siren | 2.74 (1.81) | 7.09 (1.72) |
| HELS | 711 | Siren sounds | 2.18 (1.39) | 7.35 (2.13) |
|  | 712 | Loud buzzer | 2.35 (1.64) | 8.20 (1.48) |
|  | 730 | Glass breaking | 2.85 (1.09) | 6.39 (1.54) |
|  | 732 | Household items crashing | 2.45 (1.44) | 7.03 (1.80) |
|  | **Average ratings** | | **2.38 (1.40)** | **7.33 (1.69)** |
|  | 224 | Children playing | 6.11 (1.90) | 5.64 (1.89) |
|  | 225 | Humming and clapping | 5.96 (1.51) | 4.83 (1.93) |
|  | 262 | Person yawning | 5.26 (1.58) | 2.88 (1.74) |
|  | 361 | Restaurant background talk | 5.36 (1.62) | 5.01 (1.65) |
| LEHS | 364 | Bar background talking | 5.19 (1.85) | 5.62 (1.75) |
|  | 365 | People laughing at a party | 6.97 (1.90) | 6.32 (1.90) |
|  | 368 | People talking in a crowd | 5.15 (1.33) | 4.75 (1.84) |
|  | 812 | Quiet church choir | 7.08 (1.62) | 3.36 (2.38) |
|  | **Average ratings** | | **5.89 (1.66)** | **4.80 (1.89)** |
|  | 120 | Rooster crowing | 5.20 (2.10) | 5.41 (2.13) |
|  | 130 | Pig snuffling | 4.64 (2.11) | 4.93 (1.98) |
|  | 151 | Robins chirping | 7.12 (1.56) | 4.47 (2.27) |
|  | 172 | Babbling brook | 6.62 (1.69) | 3.36 (2.07) |
| LELS | 375 | Polaroid sounds | 5.99 (1.60) | 4.48 (1.74) |
|  | 425 | Train passing by | 4.95 (1.24) | 5.20 (1.68) |
|  | 700 | Toilet flushing | 4.40 (1.52) | 4.08 (2.01) |
|  | 725 | Pouring a soda | 6.52 (1.84) | 4.26 (2.04) |
|  | **Average ratings** | | **5.68 (1.71)** | **4.52 (1.99)** |

Note: The mean and SD values presented in this table are those reported for each sound in the IADS technical report.
