# Supplementary material for: Vagal Flexibility Mediates the Association Between Resting Vagal Activity and Cognitive Performance Stability Across Varying Socioemotional Demands
Source: Front Psychol. 2020 Sep 9;11:2093. doi: 10.3389/fpsyg.2020.02093 (PMC7509204; doi:10.3389/fpsyg.2020.02093)
Supplement: Supplementary file 3 [file Data_Sheet_3.docx]

**Appendix B: Glossary**

**Consistency:** Metric of intra-individual variability in performance across experimental trials that do not appreciably differ in external task demands. Higher consistency reflects less intra-individual trial-to-trial variability in cognition over time. Lower consistency reflects more intra-individual trial-to-trial variability in cognition. Consistency is thought to be largely affected by endogenous factors such as spontaneous task-unrelated thoughts and mind-wandering.

**Dispersion:** Metric of intra-individual variability in performance across different task external demands. Higher dispersion reflects more intra-individual task-to-task variability in cognition over time. Lower dispersion reflects less intra-individual task-to-task variability in cognition. Dispersion is thought to be largely affected by exogenous factors related to varying task demands. Lower levels of dispersion are synonymous with higher levels of performance stability, our preferred term.

**Emotional distraction:** The tendency of irrelevant emotional stimuli to recruit cognitive resources, thereby interrupting performance on a separate goal-directed task.

**Interference_SD_:** Our operationalization of performance stability, reflecting the standard deviation of Stroop RT interference scores across four different Stroop tasks that varied in the type of motivational distractors. Higher scores indicate less stability (i.e., greater intra-individual variability) in cognitive inhibition performance. Lower scores indicate greater stability (i.e., less intra-individual variability in cognitive inhibition performance across task demands.

**Interference_MEAN_:** Our operationalization of performance, reflecting mean Stroop RT interference scores across four different Stroop tasks that varied in the type of motivational distractors. Higher scores indicate higher mean levels of cognitive inhibition performance across task demands. Lower scores indicate lower mean levels of cognitive inhibition performance.

**Intra-individual variability:** Individual difference construct reflecting the degree of short-term reversible change in behavior and/or physiology within an individual over time.

**Performance stability:** Individual difference construct reflecting the degree to which performance is more or less static across multiple changing contexts. Higher performance stability indexes less intra-individual variability in performance across contexts. Although different forms of performance can be probed in regard to their stability, this paper focuses on stability in inhibition performance.

**Reactivity_MEAN_:** Our operationalization of the degree of vagal reactivity across different contexts (i.e., motivational distractor conditions). This metric is computed as the mean of HRV reactivity scores across varying conditions. Individuals with higher negative scores on this metric have— on average, across all four motivational distractor conditions— stronger HRV decreases from baseline to task.

**Reactivity_SD_:** Our operationalization of individual differences in vagal flexibility. This metric computes vagal flexibility as intra-individual variability, specifically as the standard deviation of HRV reactivity scores across varying conditions. Individuals with higher scores on this metric exhibit greater differences in (HRV) vagal reactivity between the motivational distractor conditions (*greater vagal flexibility*). Individuals with lower scores on this metric exhibit weaker differences in (HRV) vagal reactivity between the motivational distractor conditions (*lower vagal flexibility*).

**Resting vagal activity:** Individual difference construct reflecting the degree of vagal activity during a resting state. This metric is thought to index trait-like capacity for adaptive behavioral, cognitive, and physiological responses across changing contexts.

**Vagal flexibility:** Individual difference construct reflecting the degree to which vagal activity is flexibly regulated across changing contexts. Higher levels of vagal flexibility represent greater intra-individual variability in vagal responses across changing contexts.

**Vagal reactivity:** Individual difference construct reflecting the degree to which vagal activity changes from baseline to task. Vagal reactivity is often assessed as change from one baseline to one stressful task, or as mean baseline-to-task change across a few different tasks.
